# Fractures of the bilateral distal radius and scaphoid: a case report

**DOI:** 10.1186/1752-1947-2-93

**Published:** 2008-03-29

**Authors:** Korhan Ozkan, Ender Ugutmen, Koray Unay, Oğuz Poyanli, Melih Guven, Abdullah Eren

**Affiliations:** 1 Goztepe Education and Research Hospital, Department of Orthopedics and Traumatology, Istanbul, Turkey

## Abstract

**Introduction:**

Bilateral fractures of the distal radius and scaphoid are extremely rare injuries.

**Case presentation:**

A patient with bilateral comminuted, displaced distal fractures of the radius and bilateral fractures of the scaphoid was treated via internal fixation of the scaphoid fractures with Herbert screws and internal fixation of the distal radius fractures with locked volar plating.

**Conclusion:**

Rigid internal fixation of distal radius and scaphoid fractures is mandatory to start early active rehabilitation of the wrist without the need for wrist immobilization with a plaster or external skeletal fixation.

## Introduction

Bilateral fractures of the distal radius and scaphoid are extremely rare injuries. In fact, we have found only one case reported in the English language medical literature; the patient had been treated using plaster immobilization [1]. In this paper, we report the case of a young man who sustained high-energy, unstable, displaced distal radius fractures along with displaced scaphoid fractures. The latter were treated with Herbert screw fixation and the former with locked volar plates. The purpose of this paper is to report the operative technique used to ensure that early wrist rehabilitation program could be started in this unusual case.

## Case presentation

**Figure 1:**
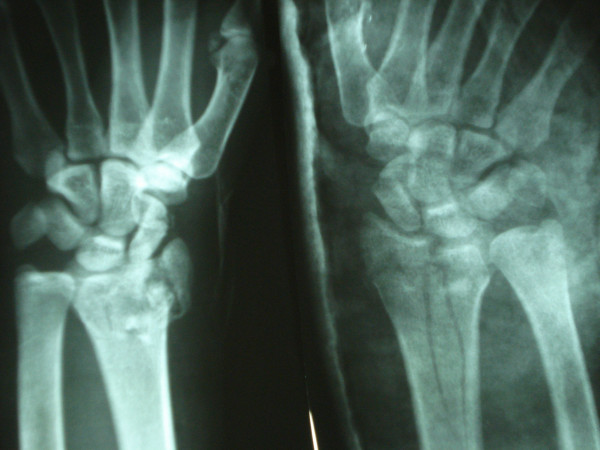
Preoperative AP roentgenogram of the right and left distal radius and scaphoid fractures.

A 28-year-old man fell from a height while working as a construction laborer. Roentgenograms displayed combined bilateral fractures of the scaphoid and distal radius. The scaphoid fractures were type B according to the Herbert classification system, and the distal radial fractures were type C according to the AO classification system (Figure 1).

The patient also sustained an anterior compression fracture of the L1 vertebrae. Open reduction of the intra-articular distal radius fractures and scaphoid fractures was performed under general anesthesia. A dissection was made between the flexor carpi radialis and palmaris longus tendons, and it was extended 3 cm distal to the wrist flexion crease to expose the scaphoid. The flexor pollicis longus tendon was retracted in the direction of the radius, while the median nerve and other tendons were retracted in the direction of the ulna, revealing the pronator quadratus. Next, the distal and radial borders of the pronator quadratus were raised and retracted in the direction of the ulna to expose the distal radius. First, the scaphoid fracture was fixed with a Herbert screw; next, open reduction of the distal radius was performed with the aid of intrafocal leverage achieved via elevation, traction, and fixation using temporary Kirschner wires. The entry site for the Herbert screw at the distal pole of the left scaphoid was comminuted, and to gain stable screw purchase, the Herbert screw was inserted from the palmar proximal toward the dorsal distal, which is a relatively infrequent procedure. No cast immobilization or bracing was used after the surgery. The patient began passive and active range of motion exercises immediately.

**Figure 2:**
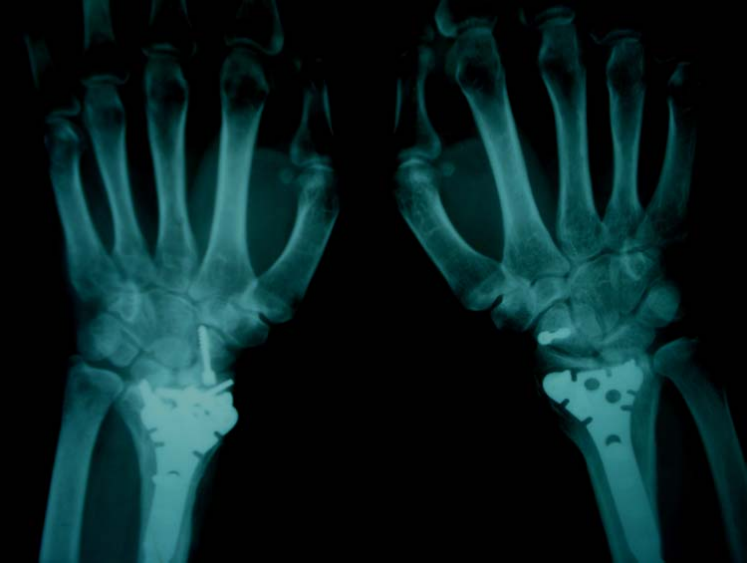
Postoperative AP roentgenogram of the right and left distal radius and scaphoid at 3 months.

**Figure 3:**
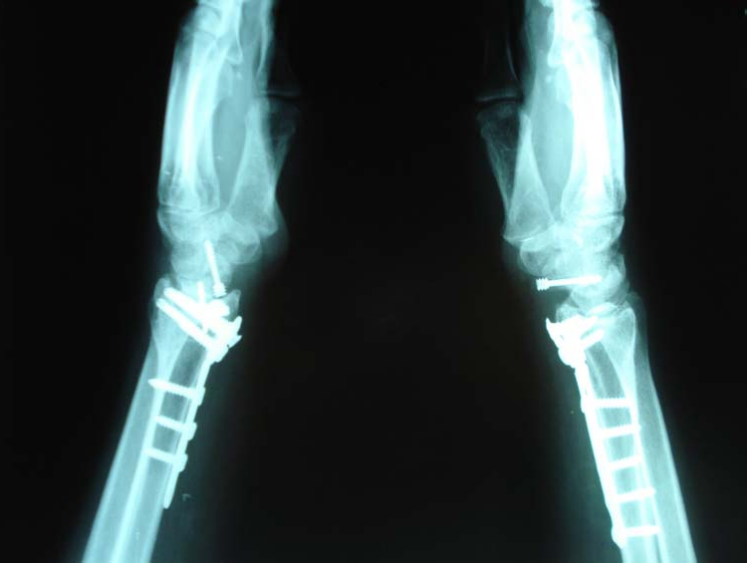
Postoperative lateral roentgenogram of the right and left distal radius and scaphoid at 3 months.

Finally, the distal radius fractures were fixed with locked volar plates. The results of roentgenographic examination conducted 3 months post injury demonstrated complete union of the scaphoid and distal radius fractures (Figures 2, 3). At 9 months after the injury, the range of wrist motion on the right side was 45° extension to 50° flexion, 20° ulnar deviation and 10° radial deviation, with 80° pronation and 70° supination; that on the left side was 40° extension to 40° flexion, 15° ulnar deviation and 10° radial deviation, with 70° pronation and 70° supination. The L1 compression fracture was treated conservatively. The patient was able to resume work at 3 months post injury.

## Discussion

Ipsilateral fractures of the distal radius and scaphoid are common injuries; however, thus far, there is only one reported case of bilateral fractures of the distal radius and scaphoid and in that case the patient was treated using plaster immobilization. Conservative management like cast immobilization may be applied in children but reduction maneuvers for distal radial fractures should be done carefully to avoid displacement of the scaphoid fracture [2,3]. Although the presence of displaced scaphoid and radius fractures in adults as in our case is an indication for operative treatment, keeping in mind that traction would be applied to the carpus to treat an unstable distal radial fracture, the presence of even an undisplaced scaphoid fracture with a displaced distal radius fracture is an indication for internal fixation of the scaphoid [4]. The three main management methods for unstable distal radial fractures are external fixation, dorsal plating, and volar plating [5].

The volar approach is advantageous to dorsal dissection, which may lead to inadequate blood supply to the dorsal metaphyseal area of the radius, can be avoided; further, this approach causes fewer problems related to the soft tissue and tendons [5,6]. The locked compression plate uses threaded screws that lock into the plate holes when tightened; this provides angular and axial stability with minimal possibility of screw loosening. In addition, these volar locking compression plates have significant strength advantages over those used in dorsal plating [5-7].

## Conclusion

High-energy traumas to the hand and wrist can result in ipsilateral and even bilateral fractures of the radius and scaphoid, and initiation of an early rehabilitation program requires rigid fixation of both these fractures. Volar locking plating of distal radius fractures and Herbert screw fixation of scaphoid fractures allow this rigid fixation but primary definitive fixation of the scaphoid, as in our case, does not allow for correction of a malalignment of the carpus following the reduction of the distal radius; therefore, temporary K-wire fixation of the scaphoid is recommended as the first step, following which screwing is done after the fixation of the distal radius, especially in the case of a preoperative carpus malalignment.

## Competing interests

The author(s) declare that they have no competing interests.

## Authors' contributions

KO and EU contributed to manuscript conception and design, carried out the literature research, manuscript preparation and manuscript review. KU and OP contributed to manuscript preparation and manuscript review. MG contributed to manuscript conception and design. AE revised the manuscript for important intellectual content.

## Consent

Written informed consent was obtained from the patient for publication of the study and any accompanying images. A copy of the written consent is available for review by the Editor-in-Chief of this journal.
